# Operative Treatment of Ankle Fractures: Predictive Factors Affecting Outcome

**DOI:** 10.7759/cureus.11016

**Published:** 2020-10-18

**Authors:** Jorge De las Heras Romero, Ana Lledó Alvarez, Carmen Torres Sánchez, Aurelio Luna Maldonado

**Affiliations:** 1 Trauma and Orthopaedics, University General Hospital Reina Sofía, Murcia, ESP; 2 Regional Statistical Center, Treasury and Public Administration Council, Murcia, ESP; 3 Forensic & Legal Medicine, University of Murcia School of Medicine, Murcia, ESP

**Keywords:** aofas, complication, injury, fracture, short form 36, surgery, malleolar, ankle and foot, open reduction internal fixation, plate

## Abstract

Purpose

Surgical management of ankle fractures has been extensively studied in literature but studies investigating validated clinical results are lacking and controversial. The purpose of this study was to evaluate functional and health-related quality of life (HRQL) outcomes after surgically treated ankle fractures and to detect some of their predictors.

Methods

Two hundred sixty-six skeletally mature patients who underwent surgery for an isolated ankle fracture from 2006 to 2017 were retrospectively identified from our hospital records and included in the study. All patients were evaluated at one, three, six and 12 months post-injury with clinical and radiographic examination. Outcome measures recorded at final two years minimum follow-up included patient-reported pain, patient satisfaction, functional (American Orthopaedic Foot & Ankle Society (AOFAS) ankle-hindfoot score) and quality of life (Short Form (SF) 36 score) status.

Results

Patient satisfaction score was 8.4 out of 10, Visual Analogue Scale mean score was 2.3, complication rate was 36.5% and mean AOFAS ankle-hindfoot score was 87.3. Mean SF36-physical summary score was 77.9 and SF36-mental summary score was 81.1. The injury demonstrated a significant effect on physical function, role-physical, bodily pain and social functioning SF-36 subdomains. Functional outcome was significantly related to occupation, syndesmotic lesion, number of fractured malleoli and delay to surgery. Main predictors of quality of life were age, occupation, cause of injury, syndesmotic lesion, number of fractured malleoli and waiting time to surgery.

Conclusions

Ankle fractures have a considerable impact on functional and quality of life status of patients. Occupation, presence of syndesmotic injury, Pott's classification and surgery delay must be considered as predictors of final outcome.

## Introduction

Ankle fractures are the most frequent fractures seen at the major trauma centers, as far as foot and ankle injuries are concerned. In addition, they account for 10% of all fractures (second in frequency behind proximal femoral fractures) and are estimated to have an incidence of about 184/100,000/year in the USA [1]. Moreover, the prevalence and incidence of these lesions have increased within the last three decades, especially in young men and elderly women, with a mean age of 46 years, significantly higher than the age of isolated ankle sprains. Furthermore, it is anticipated that the number of these lesions could be expected to triple by 2023 [2].

Although these fractures have traditionally been considered to have a good prognosis, more recent studies suggest that between 17% and 24% of patients may not have a satisfactory outcome [3].

Additionally, there are very few studies evaluating predictors of both health-related quality of life (HRQL) and functional outcomes after a surgically treated ankle fracture. Moreover, in many cases their interpretation is limited due to methodological problems, such as poor sample selection, high loss of follow-up, inability to describe the predictive value of individual variables or combinations of variables or the use of a non-validated measure for HRQL and functional outcomes.

The purpose of this study is to evaluate the effect of ankle fractures treated surgically on the HRQL, functional status and pain score, as measured by the validated Short Form 36 health survey (SF-36), the American Orthopaedic Foot and Ankle Society (AOFAS) ankle-hindfoot score and Visual Analogue Scale (VAS), respectively. Furthermore, we aim to identify prognostic factors that influence these outcomes. Good knowledge of these variables would be very useful for clinical practice, not only to identify patients at risk of poor outcomes but also to allocate more resources to their postoperative management.

## Materials and methods

This study retrospectively included all patients who underwent surgery for an ankle fracture performed by consultants in our center from January 2006 to April 2017 and met the following inclusion criteria: skeletally mature patients, malleolar ankle fracture AO/OTA 4 type 44 [4], displaced and surgically treated, and a minimum follow-up of two years. Patients with multiple fractures were excluded.

Data from patients’ records were collected including 1) age; 2) gender; 3) occupation; 4) mechanisms of injury; 5) impact energy; 6) comorbidities; 7) fracture classification; 8) time to surgery; 9) hospital stay; 10) duration of immobilization and 11) complications. All patients had routine clinical and radiological assessments performed preoperatively and at one, three, six and 12 months after injury. In addition, all patients were reviewed once post follow-up for completion of outcome forms. The collection of data was performed by an independent observer who was not involved in treatment. Fractures were classified according to AO/OTA classification [4], Gustilo grading system [5], Pott classification [6] and a new classification introduced by the corresponding author (DL Heras) not yet published in relation to the injury of the syndesmosis (no lesion of the inferior tibiofibular ligament, partial lesion and complete lesion) determined during surgery. Early complications were the ones that occurred just after the injury or during initial treatment, and late complications were present at final follow-up. Range of ankle mobility was measured with a goniometer at final follow-up. Standard radiographs (anteroposterior, lateral and mortise views) were performed at each postoperative consultation. The fracture union was defined as the presence of bridged callus in at least three of four cortices on two orthogonal radiographic views, delayed union as no callus in the first three months and nonunion as no callus within six months.

At final follow-up, the first outcome considered in this study was HRQL as measured by the Short Form-36 (SF36) health survey validated for our country [7]. Results were divided into two components (physical and mental component summaries), and transformed to range 0 (worst quality of life) to 100 (best quality of life). These data were compared with those in the national standard for the gender and age-matched general population [8]. Moreover, SF-36 values were considered as satisfactory or unsatisfactory if they were greater or less than 80 points. Ponzer et al. [9] concluded that this form is widely accepted as a generic outcome measure, being, a useful tool to assess outcomes after ankle fractures [10]. Functional assessment of the patient was recorded by means of the 0-100 AOFAS ankle-hindfoot score [8], with 0 indicating worst clinical condition and 100 indicating best clinical condition. Outcome was classified as "fair", at least (greater than 60 points) or "poor" (60 or less) [10,11]. Patient satisfaction score was measured on a 0-10 scale (completely satisfied-completely dissatisfied). Finally, level of pain was also noted as measured by the 0-10 VAS (no pain-maximum pain).

Statistical analysis

All data were recorded on a computerized data collection form (Excel version 2013) and statistical analysis was performed using SPSS software version 20.0 (IBM Corp., Armonk, NY). Normality was tested by the Kolmogorov-Smirnov test. For univariate analysis, chi-square with Yates correction, t-Student and ANOVA tests were used. Multiple logistic regression was performed to analyze the influence of covariates on outcomes at last follow-up. Separate regression models were constructed for each SF36 component summaries and for the AOFAS ankle-hindfoot score. Only those variables that were statistically significant in univariate analysis (p < 0.05) were included in multivariate analysis. Data were presented as adjusted risks (OR: Odds ratio) with 95% confidence intervals (CI). In all cases, statistical significance was considered for p-values less than 0.05.

A posteriori statistical power was calculated by estimation of relative risk in the cohort study of 266 patients. Using an estimated fracture prevalence of thirty per thousand (samples ratio of 0.030), assuming a standardized mean difference of 0.15 (clinically relevant difference of 3.5 points in health-related quality of life), and a confidence level of 0.05, a power of 80% was obtained.

## Results

Two hundred seventy cases were initially included in the study, of which four were excluded because they were patients with multiple trauma. Of the 266 patients who were eligible, 121 (46%) were men and 145 (54%) women with a mean age at injury of 48.6 ± 18 (range, 16-87) years. Sixty-nine percent of the patients aged less than 60 years. The mean follow-up was 83 ± 40 (range, 24-151) months and the average patient satisfaction score was 8.4 ± 2 on a 0-10 scale (range, 2-10).

According to the AO/OTA classification, there were 16 type-44 A or infrasyndesmotic (6%), 214 type-44 B or transsyndesmotic (80%) and 36 type-44 C or suprasyndesmotic (14%) fractures. Regarding Pott classification, there were 107 type-A (42%), 126 type-B (47%) and 33 type-C (13%) fractures. According to Gustilo classification, nine fractures were open, three grade I, three grade II and three grade III. At the time of injury, 20 patients were self-employed, 73 employees, 85 unemployed and 88 pensioners. The mechanisms of injury included 207 falls, 29 traffic accidents, 20 sports injuries, five workplace injuries and five assaults. A total of 241 patients had low energy and 25 high energy injury. Twenty-five patients suffered from osteoporosis and 21 diabetes. In all patients open reduction and internal fixation (ORIF) was performed when the skin's condition was acceptable, at a mean of 2.7 ± 3.2 (range, 0-16) days. Furthermore, mean hospital stay was 5.4 ± 4.7 (range, 1-54) days and mean ankle immobilization time was 29.8 ± 8.2 (range, 13-66) days. VAS mean score was 2.3 ± 1.8 (range, 0-8) points.

There were short-term complications in 59 patients (22.2%), including 14 cases of complex regional pain syndrome (mean 42 ± 5.4), three cases of osteochondral lesion of the talus, seven cases of superficial wound infection treated with antibiotics, 20 cases of skin necrosis, four cases of deep infection resolved without difficulty after debridement and removal of infected material, five cases of pressure ulcer, four cases of implant failure treated surgically afterwards and one case of amputation neuroma. There was a delayed union in 10 patients and nonunion in three (bone finally healed after subsequent surgery in all cases). Long-term complications were present in 74 patients (27.8%) such as chronic pain (41 cases), joint stiffness (22 cases), osteoarthritis (nine cases) and instability (two cases).

Health-related quality of life outcomes

The SF-36 is a validated general health measure for orthopaedic conditions and the standard against which many instruments are measured in validation studies. It will be used in this study to measure quality of life outcomes.

At final follow-up, the mean SF36-physical summary score was 77.9 ± 18 (range, 15-95) and SF36-mental summary score was 81.1 ± 17.6 (range, 17-98) points, in total 62.8% and 66.9% with satisfactory outcomes, respectively. Injury did not demonstrate a significant effect on general health, vitality, role-emotional and mental health subscores compared to those parameters in gender and age-matched national normative data. In contrast, physical function, role-physical, bodily pain and social functioning domains remained significantly lower than country norms (p = 0.000) (Table 1).

**Table 1:** SF-36 subscores in the study group against age-matched country norms. *Data are shown as average (standard deviation). †Two-tailed Student’s t test.

	Fracture*	National Norms*	p-value†
Physical Function	85.1 (18.2)	90.3 (17.1)	0.000
Role Physical	79.2 (20)	84.3 (15.8)	0.000
Bodily Pain	75.8 (17.7)	81.9 (16.0)	0.000
General Health	71.5 (17.1)	70.9 (19.6)	0.000
Vitality	71.4 (20)	71.8 (21.0)	0.776
Social Functioning	86.1 (19.2)	94.1 (16.6)	0.000
Role Emotional	88.9 (16.7)	90.3 (18.2)	0.186
Mental Health	78 (15.8)	77.9 (18.7)	0.884
Physical Summary	77.9 (18)	81.9 (16.4)	0.000
Mental Summary	81.1 (17.6)	83.6 (18.5)	0.021

As seen in Table 2, predictors of physical summary scores following ankle fractures were: age, occupation, mechanisms of injury, impact energy, Pott and Romero classifications, time to surgery and short-term complications. SF36-physical outcome was not associated with gender (p = 0.165), comorbidities (p = 0.462), AO/OTA classification (p = 0.476), Gustilo classification (p = 0.396), hospital stay (p = 0.656) or duration of immobilization (p = 0.197). Likewise, strong significant association was found between SF36 physical component summary and AOFAS ankle-hindfoot score (p = 0.000).

**Table 2:** Factors predicting SF-36 physical summary score. SD: Standard deviation; CI: Confidence interval; OR: Odds ratio; Ref: Reference value; Syndes: Syndesmotic injury.

	Bivariate			Multivariate	
	n = 266	Mean (SD)		OR (95% IC)	
Age			p		p
≤50 years	143	81 (14.5)		Ref.	
>50 years	123	74.2 (20.8)	0.002	2.1 (1.3-3.4)	0.005
Occupation					
Employee	73	81.6 (12.1)		Ref.	
Unemployed	85	79.2 (17.1)		1.07 (0.5-1.9)	0.882
Pensioner	88	76.6 (19.7)		1.3 (0.7-2.6)	0.367
Self-employed	20	64.5 (24.9)	0.002	3.8 (1.3-10.7)	0.012
Cause of injury					
Fall or sports	227	78.9		Ref.	
Other	39	72.3	0.036	2.2 (1.1-4.4)	0.022
Impact energy					
Low	241	78.8 (17.8)		Ref.	
High	25	69.2 (17.9)	0.011	3.4 (1.4-8)	0.005
Pott type					
I	107	81.1 (14.8)		Ref.	
II	126	76.9 (19.2)		2 (1.2-3.6)	0.012
III	33	71.1 (20.5)	0.015	3.4 (1.5-7.6)	0.003
Heras type					
No syndes.	150	80.4 (16.2)		Ref.	
Partial syndes.	83	75 (18.8)		2.1 (1.2-3.7)	0.008
Complete syndes.	33	73.8 (21.7)	0.034	2.3 (1.1-4.9)	0.037
Time to surgery					
>1 day	132	80.6 (15.8)		Ref.	
≤1 day	134	75.2 (19.5)	0.015	1.8 (1.1-3)	0.021
Early complications					
No	202	81.4 (15.2)		Ref.	
Yes	64	66.8 (21.4)	0.000	4.9 (2.7-8.8)	0.000

Regarding SF-36 mental summary scores, Table 3 demonstrates same predictors except impact energy and short-term complications. Therefore, SF36-mental outcome was not associated with gender (p = 0.129), impact energy (p = 0.138), comorbidities (p = 0.492), AO/OTA classification (p = 0.467), Gustilo classification (p = 0.520), hospital stay (p = 0.673), duration of immobilization (p = 0.141) or short-term complications (p = 0.778). Likewise, strong significant relationship was found between SF36 mental component summary and AOFAS ankle-hindfoot score (p = 0.000).

**Table 3:** Factors predicting SF-36 mental summary score. SD: Standard deviation; CI: Confidence interval; OR: Odds ratio; Ref: Reference value; Syndes: Syndesmotic injury.

	Bivariate		Multivariate		
	n = 266	Mean (SD)		OR (95% IC)	
Age			p		p
≤50 years	143	84.2 (13.8)		Ref.	
>50 years	123	77.4 (20.5)	0.001	1.9 (1.1-3.2)	0.016
Occupation					
Employee	73	85 (11.6)		Ref.	
Unemployed	85	82.2 (16.7)		1.02 (0.5-2)	0.951
Pensioner	88	79.7 (19.3)		1.1 (0.6-2.2)	0.702
Self-employed	20	68.3 (24.3)	0.001	2.8 (1.03-7.8)	0.044
Cause of injury					
Fall or sports	227	82		Ref.	
Other	39	75.7	0.038	2.5 (1.2-4.9)	0.010
Pott type					
I	107	84.2 (14.3)		Ref.	
II	126	80.2 (18.8)		2.1 (1.2-3.8)	0.011
III	33	74.5 (20.2)	0.016	3.2 (1.4-7.4)	0.005
Heras type					
No syndes.	150	83.5 (15.9)		Ref.	
Partial syndes.	83	78.3 (18.1)		2.3 (1.3-4.1)	0.004
Complete syndes.	33	77.1 (21.8)	0.035	2.5 (1.2-5.5)	0.019
Time to surgery					
>1 day	132	80.6 (15.8)		Ref.	
≤1 day	134	75.2 (19.5)	0.015	1.8 (1.1-3)	0.021

Functional outcomes

AOFAS score is the most popular outcome measure in articles focused on ankle fractures [10]. It will be used in the present study to measure functional outcomes.

At the final follow-up, the mean AOFAS ankle-hindfoot score was 87.3 ± 18.2 (range, 22-100) points, in total 85.3% with satisfactory outcome. As seen in Table 4, predictors were occupation, Pott and Heras classification systems and delay to surgery; therefore the non-associated independent variables were age (p = 0.392), gender (p = 0.125), mechanisms of injury (p = 0.086), impact energy (p = 0.096), comorbidities (p = 0.588), AO/OTA classification (p = 0.343), Gustilo classification (p = 0.203), hospital stay (p = 0.378), duration of immobilization (p = 0.167) and short-term complications (p = 0.818).

**Table 4:** Factors predicting AOFAS ankle-hindfoot score. SD: Standard deviation; CI: Confidence interval; OR: Odds ratio; Ref: Reference value; Syndes: Syndesmotic injury; AOFAS: American Orthopaedic Foot and Ankle Society.

Bivariate				Multivariate	
	n = 266	Mean (SD)	p	OR (95% IC)	p
Occupation					
Employee	73	91.2 (12.8)		Ref.	
Unemployed	85	88.2 (17.3)		2.2 (0.7-6.7)	0.150
Pensioner	88	85.5 (20.1)		2.8 (0.9-8.1)	0.059
Self-employed	20	76.4 (25.1)	0.009	7.3 (2-26.6)	0.003
Pott type					
I	107	91 (14.1)		Ref.	
II	126	85.8 (20)		2.9 (1.2-6.8)	0.013
III	33	80.6 (20.5)	0.007	3.3 (1.1-10)	0.032
Heras type					
No syndes.	150	90 (16.1)		Ref.	
Partial syndes.	83	84 (19.2)		1.8 (0.9-4)	0.115
Complete syndes.	33	82.9 (22.3)	0.016	2.7 (1.04-6.9)	0.042
Time to surgery					
>1 day	132	90.6 (15.1)		Ref.	
≤1 day	134	84 (20.4)	0.003	3.4 (1.6-7.2)	0.002

## Discussion

It is increasingly thought that ankle fractures have important implications on function or quality of life. The impact of these lesions is not only restricted to pain and functional impairment caused at the time of injury, but it can also frequently lead to prolonged periods of immobilization and functional limitation, the inability to perform regular activities in the medium to long term or other sequelae, sometimes in spite of receiving correct surgical treatment. Despite this, sustained outcomes still remain unclear because researchers report conflicting results on many occasions.

Ankle fractures carry an inherent risk of complications. The overall complication rate following surgical treatment of ankle fractures varies considerably in the literature ranging from 1% to 40%. SooHoo et al. [12] reported a low overall rate of short-term complications below 5% after surgical treatment of this injuries in a population of 57,183 patients, using the California’s discharge database. Mak et al. [13] studied 116 cases and noted a rate of local complications of 11.6%. Other authors noted much higher complication rates [14,15]. These differences may be explained by heterogeneity of studies in relation to the population, type of fracture or complication included. In the current study, the overall complication rate was 36.5%.

Functional outcome after surgery of an ankle fracture has also been reported. Verhage et al. [16] conducted a retrospective cohort-study on 243 operated malleolar fractures and resulted in an overall AOFAS score of 95 after a mean follow-up of 9.6 years. Noh et al. [17] reported an average AOFAS score of 85, in a prospective randomized study including 109 subjects with an ankle fracture. In the current study, mean AOFAS ankle-hindfoot score was 87, in line with previous literature.

Clinical outcomes and complications have been widely studied but very few have analyzed HRQL after these fractures. The most frequently used measure of HRQL in the literature is the SF-36 scale. Bhandari et al. reported physical function and role-physical subscores to be significantly lower than US norms at 24 months after operative treatment of 30 patients with unstable ankle fractures in a prospective observational cohort study [3]. In contrast Obremskey et al. [18], in a 20 patients’ study with isolated unstable ankle fracture treated surgically, only found significant differences in the physical function subdomain after a 20-month follow-up, and Nilsson et al. [19], studying 60 patients in a case-series descriptive study found no differences after one-year follow-up. In the present study, physical function, role-physical, bodily pain, social functioning and both physical and mental summary domains of the SF36 were significantly lower in patients with surgically treated ankle fracture compared to the standard of the healthy population in our country. In line with previous studies our data confirm that these injuries decrease the quality of life of patients, mainly their physical capacity.

Very few researchers have looked at factors that may predict short-term functional outcome following ORIF of ankle fractures. In a prospective cohort study performed by Hancock et al. [20] on 62 consecutive patients with ankle fracture, ankle dorsiflexion measured at the time of cast removal and fracture Pott’s classification were reported as clinically significant predictors. Egol et al. [21] reviewed retrospectively 347 patients and concluded that those who required syndesmotic stabilization had poorer outcomes at 12 months. Egol et al. also reported that patient age was not predictive of functional recovery at one year. Cavo et al. [22], using data from the Nationwide Inpatient Sample, collected 148,483 patients with ankle fracture and identified diabetes and obesity as predictors of higher health-care utilization and costs. Lanzetti et al. [23] concluded that diabetes mellitus and higher BMI delay wound healing and increase complication rate in young adult patients with surgically treated bimalleolar fractures. Bhandari et al. also included smoking history, presence of medial malleolar fracture and lower level of education as significant independent predictors [3]. Similarly, Dodson et al. found smoking, obesity and diabetes as factors linked to the prognosis, although they did not use a recognized outcome measure. Böstman [24] identified overweight as a significant factor for loss of reduction in a series of 3061 patients with ankle fracture, mostly treated by ORIF. Menendez et al. [25] determined that patients with metabolic syndrome (hypertension, diabetes, dyslipidemia or obesity) were at increased risk for posthospitalization care. Alcoholism was also documented as a predictor of complications in a retrospective study performed by Tønnesen et al. [26]. Jones et al. [27] also found an increased risk of complications in diabetic patients in a retrospective case-control study. Another study by Black et al. [28] found that early weight-bearing could allow earlier return to work. Despite all this literature, we consider that only few predictors are known and most of previous studies do not use recognized outcome measures. In the current study, we identified age >50 years, occupation (self-employed), cause of injury other than fall or sports, syndesmotic lesion, greater number of fractured malleoli and delay time to surgery <1 day as significant predictors for HRQL unsatisfactory outcome. We also identified same factors except age and cause of injury as significant predictors for functional outcome behaving in the same manner. Although current literature supports that ankle fractures surgery should be performed within 7 to 24 hours of injury or up to 12 days after injury until soft tissue swelling has settled to avoid complications, we believe that in our hospital deferred non-urgent surgery yielded better results, since we have specific trained personnel. Gender, comorbidities, Gustilo and AO/OTA classifications, hospital stay or duration of immobilization were not found to be significant predictors. These variables may lead to an increase in complications in these fractures, as reflected in the aforementioned literature, but in our study they do not seem to influence the final result of health-related or functional scales.

In the present study, and so far not investigated in any previous study, there was a strong significant association between ankle functional outcomes measured with AOFAS ankle-hindfoot score and those referred to quality of life measured with SF-36. This seems to indicate the predictive value of AOFAS score on the prognosis of quality of life.

This study had certain limitations. The retrospective design of the study could not allow the analysis of some other variables and no comparative data were available on the preinjury status. Another limitation was the relatively short follow-up. Although the minimum of two years post-trauma is met and that some authors even reported that stabilization of outcomes occurs before the year after injury [29-30], we would have preferred a minimum follow-up period of at least three years to confirm residual instability due to ligaments damage at the moment of the injury or posttraumatic osteoarthritis due to cartilage damage.

The authors recommend that future studies should measure validated patient-reported outcomes including HRQL and functional tests similar to present work. We believe that accurate estimation of HRQL or functional outcomes is important for several reasons: 1) prognostic estimates can guide the patient’s expectations after this injury and also may be useful in considering medicolegal and workers’ compensation issues; 2) a physician can use this estimates as a guide for ordering additional tests and selecting appropriate therapies and, finally, 3) knowledge of predictive factors can be useful in designing future randomized clinical trials.

Correlation between AOFAS ankle-hindfoot score and SF-36 should be further investigated through studies with larger samples. If its association is confirmed, AOFAS scale could also be used to analyze quality of life if validated.

Finally, we also recommend further studies analyzing outcomes of bimalleolar equivalent fractures with lesion of the deltoid ligament, Maisonneuve fractures, or malleolar fractures that involve, e.g., lateral and posterior malleoli, as we could anticipate worse outcome.

## Conclusions

Ankle functional outcome after a malleolar ankle fracture was significantly related to occupation, syndesmotic lesion, number of fractured malleoli and delay time to surgery. The main predictors of HRQL were age, occupation, cause of injury, syndesmotic lesion, number of fractured malleoli and delay time to surgery. AOFAS ankle-hindfoot score was strongly associated to SF36 score. Physical function, role-physical, bodily pain and social functioning SF-36 subdomains were significantly below the mean normal population-based norms.

Ankle fractures have a considerable impact on functional and quality of life status of patients. Occupation, presence of syndesmotic injury, number of fractured malleoli (Pott classification) and delay time to surgery must be considered as predictors of final ankle functional and HRQL outcomes.

## References

[1] Shibuya N, Davis ML, Jupiter DC (2014). Epidemiology of foot and ankle fractures in the United States: an analysis of the National Trauma Data Bank (2007 to 2011). J Foot Ankle Surg.

[2] Kannus P, Palvanen M, Niemi S, Parkkari J, Järvinen M (2002). Increasing number and incidence of low-trauma ankle fractures in elderly people: Finnish statistics during 1970-2000 and projections for the future. Bone.

[3] Bhandari M, Sprague S, Hanson B, Busse JW, Dawe DE, Moro JK, Guyatt GH (2004). Health-related quality of life following operative treatment of unstable ankle fractures: a prospective observational study. J Orthop Trauma.

[4] Marsh JL, Slongo TF, Agel J (2007). Fracture and dislocation classification compendium - 2007: Orthopaedic Trauma Association classification, database and outcomes committee. J Orthop Trauma.

[5] Gustilo RB, Anderson JT (2002). Prevention of infection in the treatment of one thousand and twenty-five open fractures of long bones: retrospective and prospective analyses. J Bone Joint Surg Am.

[6] Pott P (2007). Some few general remarks on fractures and dislocations. Clin Orthop Relat Res.

[7] Alonso J, Prieto L, Anto JM (1995). La versión española del SF-36 Health Survey (Cuestionario de Salud SF- 36): un instrumento para la medida de los resultados clínicos. Med Clin.

[8] Alonso J, Regidor E, Barrio G, Prieto L, Rodríguez C, de la Fuente L (1998). Population reference values of the Spanish version of the Health Questionnaire SF-36. (Article in Spanish). Med Clin.

[9] Ponzer S, Nåsell H, Bergman B, Törnkvist H (1999). Functional outcome and quality of life patients with type B ankle fractures: a two-year follow-up study. J Orthop Trauma.

[10] Hunt KJ, Hurwit D (2013). Use of patient-reported outcome measures in foot and ankle research. J Bone Joint Surg Am.

[11] Kitaoka HB, Alexander IJ, Adelaar RS, Nunley JA, Myerson MS, Sanders M (1994). Clinical rating systems for the ankle-hindfoot, midfoot, hallux, and lesser toes. Foot Ankle Int.

[12] SooHoo NF, Krenek L, Eagan MJ, Gurbani B, Ko CY, Zingmond DS (2009). Complication rates following open reduction and internal fixation of ankle fractures. J Bone Joint Surg Am.

[13] Mak KH, Chan KM, Leung PC (1985). Ankle fracture treated with the AO principle: an experience with 116 cases. Injury.

[14] Zaghloul A, Haddad B, Barksfield R, Davis B (2014). Early complications of surgery in operative treatment of ankle fractures in those over 60: a review of 186 cases. Injury.

[15] Koval KJ, Zhou W, Sparks MJ, Cantu RV, Hecht P, Lurie J (2007). Complications after ankle fracture in elderly patients. Foot Ankle Int.

[16] Verhage SM, Schipper IB, Hoogendoorn JM (2015). Long-term functional and radiographic outcomes in 243 operated ankle fractures. J Foot Ankle Res.

[17] Noh JH, Roh YH, Yang BG, Kim SW, Lee JS, Oh MK (2012). Outcomes of operative treatment of unstable ankle fractures: a comparison of metallic and biodegradable implants. J Bone Joint Surg Am.

[18] Obremskey WT, Dirschl DR, Crowther JD, Craig WL 3rd, Driver RE, LeCroy MC (2002). Change over time of SF-36 functional outcomes for operatively treated unstable ankle fractures. J Orthop Trauma.

[19] Nilsson G, Jonsson K, Ekdahl C, Eneroth M (2007). Outcome and quality of life after surgically treated ankle fractures in patients 65 years or older. BMC Musculoskelet Disord.

[20] Hancock MJ, Herbert RD, Stewart M (2005). Prediction of outcome after ankle fracture. J Orthop Sports Phys Ther.

[21] Egol KA, Pahk B, Walsh M, Tejwani NC, Davidovitch RI, Koval KJ (2010). Outcome after unstable ankle fracture: effect of syndesmotic stabilization. J Orthop Trauma.

[22] Cavo MJ, Fox JP, Markert R, Laughlin RT (2015). Association between diabetes, obesity, and short-term outcomes among patients surgically treated for ankle fracture. J Bone Joint Surg Am.

[23] Lanzetti RM, Lupariello D, Venditto T, Guzzini M, Ponzo A, De Carli A, Ferretti A (2018). The role of diabetes mellitus and BMI in the surgical treatment of ankle fractures. Diabetes Metab Res Rev.

[24] Böstman OM (1995). Body-weight related to loss of reduction of fractures of the distal tibia and ankle. J Bone Joint Surg Br.

[25] Menendez ME, Neuhaus V, Bot AG, Ring D, Johnson AH (2014). The impact of metabolic syndrome on inpatient outcomes after isolated ankle fractures. Foot Ankle Int.

[26] Tønnesen H, Pedersen A, Jensen MR, Møller A, Madsen JC (1991). Ankle fractures and alcoholism. The influence of alcoholism on morbidity after malleolar fractures. J Bone Joint Surg Br.

[27] Jones KB, Maiers-Yelden KA, Marsh JL, Zimmerman MB, Estin M, Saltzman CL (2005). Ankle fractures in patients with diabetes mellitus. J Bone Joint Surg Br.

[28] Black JD, Bhavikatti M, Al-Hadithy N, Hakmi A, Kitson J (2013). Early weight-bearing in operatively fixed ankle fractures: a systematic review. Foot.

[29] Davidovitch RI, Walsh M, Spitzer A, Egol KA (2009). Functional outcome after operatively treated ankle fractures in the elderly. Foot Ankle Int.

[30] Velleman J, Nijs S, Hoekstra H (2018). Operative management of AO type 44 ankle fractures: determinants of outcome. J Foot Ankle Surg.

